# Herbal Preparations of Medical Cannabis: A Vademecum for Prescribing Doctors

**DOI:** 10.3390/medicina56050237

**Published:** 2020-05-15

**Authors:** Pietro Brunetti, Simona Pichini, Roberta Pacifici, Francesco Paolo Busardò, Alessandro del Rio

**Affiliations:** 1 Department of Excellence of Biomedical Sciences and Public Health, “Politecnica delle Marche” University of Ancona, Via Tronto 71, 60126 Ancona, Italy; pietro.brunetti40@gmail.com; 2 Analytical Pharmacotoxicology Unit Head, National Centre on Addiction and Doping, Istituto Superiore di Sanità V.Le Regina Elena 299, 00161 Rome, Italy; simona.pichini@iss.it (S.P.); roberta.pacifici@iss.it (R.P.); 3 Department of Anatomical, Histological, Forensic and Orthopedic Sciences, Sapienza University, 00161 Rome, Italy; alessandro.delrio@uniroma1.it

**Keywords:** cannabis, medical use, herbal preparation, cannabinoids, treatment, interactions, titration

## Abstract

Cannabis has been used for centuries for therapeutic purposes. In the last century, the plant was demonized due to its high abuse liability and supposedly insufficient health benefits. However, recent decriminalization policies and new scientific evidence have increased the interest in cannabis therapeutic potential of cannabis and paved the way for the release of marketing authorizations for cannabis-based products. Although several synthetic and standardized products are currently available on the market, patients’ preferences lean towards herbal preparations, because they are easy to handle and self-administer. A literature search was conducted on multidisciplinary research databases and international agencies or institutional websites. Despite the growing popularity of medical cannabis, little data is available on the chemical composition and preparation methods of medical cannabis extracts. The authors hereby report the most common cannabis preparations, presenting their medical indications, routes of administration and recommended dosages. A practical and helpful guide for prescribing doctors is provided, including suggested posology, titration strategies and cannabinoid amounts in herbal preparations obtained from different sources of medical cannabis.

## 1. Introduction

The medical use of preparations derived from *Cannabis sativa* L. has a long history [1,2]. In Asia, mainly in China and India, cannabis has been used for centuries for religious and medical purposes to treat neuralgias, convulsions, migraines [3,4], rheumatic pain, intestinal constipation, sexual disorders [4], asthma, malaria [5] and gout [3,4,5]. From the 19th century onwards, cannabis use also became very popular in Europe and America [3], and the plant entered Western pharmacopoeias as a medical remedy [6]. Tinctures and other preparations were used to treat various disorders including convulsions in infants, tetanus, migraine, enteralgia, neuralgia, insomnia and hysteria [3,7]. However, due to the inability to prepare standardized preparations and the diffusion of the recreational use of cannabis below therapeutic concentrations from the end of the 19th to the first half of the 20th century [3], medical use of cannabis began to decline [7]. In the United States, the “Marihuana Tax Act″, a restrictive federal legislation, functionally ended all medical uses of cannabis in 1937 [8], and cannabis was removed from the “National Formulary and Pharmacopoeia″ in 1941 [9]. In 1961, cannabis, including resin, extracts and tinctures, was listed in the Schedule I of the Single Convention on Narcotic Drugs [1], which prohibits the use, possession, production, manufacture, export, import, distribution and trade of cannabis and other drugs except for medical and scientific purposes [10].

During the 1960s, the most abundant and main active ingredients of *Cannabis sativa* L., delta-9-tetrahydrocannabinol (THC) and cannabidiol (CBD), were characterized and described by Mechoulam et al. [11,12,13,14]. As the main psychotropic compound of cannabis, THC was considered to represent a substantial risk to public health, with small to moderate therapeutic effects, and was listed in schedule II of the Convention of Psychotropic Substances of 1971 [15]. Nevertheless, in 1985, the United States Food and Drug Administration (US FDA) reconsidered the medical use of cannabinoids and THC, approving Marinol^®^ (dronabinol) and Cesamet^®^ (nabilone), two synthetic analogues of THC, for the management of nausea and vomiting associated with cancer chemotherapy in patients who did not respond to conventional antiemetic treatments [16,17]. The interest in the potential medical use of cannabis and cannabinoids significantly rose in the 1990s, following the discovery of the endocannabinoid (eCB) system in mammals; this was considered as a way of inducing analgesia and providing therapeutic strategies for treating different forms of chronic pain [18,19,20,21,22,23,24] and neurological disorders such as multiple sclerosis [21,22], epilepsy [23,24] and other movement disorders [19,25,26]. The possibility to exploit herbal cannabis and cannabinoids (natural or synthetic ones) for medical and/or scientific purposes, together with the latest cannabis decriminalization polices [27,28,29], paved the way for the release of licenses and marketing authorizations for products containing or derived from cannabis and cannabinoids. In 1999, the FDA also approved Marinol^®^ for the treatment of anorexia associated with weight loss in patients with Acquired Immune Deficiency Syndrome (AIDS) [16]. Over the next twenty years, other synthetic products obtained FDA market authorization, including Syndros^®^ (dronabinol, oral solution) and Canemes^®^ (nabilone, capsules), and many studies focused on discovering synthetic analogues possessing high pharmacological activity with less psychotropic effects. In contrast, in Europe, much attention was also paid to herbal preparations, composed of cannabis female flowering tops, which can be used for decoctions, oils and sprays or simply inhaled with a vaporizer [1].

Although industrial oral sprays and vaporized forms through established devices (i.e., Volcano^®^ and pen vaporizers) [22,30] are available, homemade decoctions and pharmacy oils are currently the most widespread cannabis formulations in Europe, making standardization of preparation difficult.

In this narrative review, we aim to describe and detail the different herbal cannabis preparations currently available to treat neuropathic pain associated with different pathologies and movement disorders, such as Tourette syndrome and Parkinson’s disease. We also report cannabinoids’ extraction recovery and stability over time in aqueous and oily preparations with the objective of providing a practical guide with posology in both preparations to help medical doctors with their initial prescriptions.

## 2. Methodology

A literature search was conducted on multidisciplinary research databases such as PubMed, Scopus and Web of Science and international agencies or institutional websites including the World Health Organization (WHO), United Nations Office on Drugs and Crime (UNODC), International Conference on Harmonisation (ICH), US FDA, US Drug Enforcement Administration (DEA), US National Institute of Health (NIH), European Monitoring Centre for Drugs and Drug Addiction (EMCDDA), European Medicines Agency (EMA), and Italian Institute of Health (ISS) to identify the most relevant literature (up to March 2020).

The search terms used alone or in combination were: “herbal preparations”, “decoction”, “oil”, “medical use”, “cannabis” “cannabinoids”, “therapy”, “adverse effects” and “titration”.

Only articles written in English were selected. All articles were reviewed independently by the authors to determine their relevance in the framework of the current study.

## 3. Herbal Preparations of Medicinal Cannabis

The term “herbal cannabis” refers to dried harvested female flowering tops, which contain the highest concentrations of cannabinoids, mainly THC, CBD, cannabigerol (CBG), cannabichromene (CBC), cannabidivarin (CBDV) and tetrahydrocannabivarin (THCV), together with their non-psychoactive carboxylated forms [31,32]. Recently, it was demonstrated that tetrahydrocannabinolic acid A (THCA-A) and cannabidiolic acid (CBDA) are involved in the medical benefits of cannabis in animal models [33,34]. Moreover, it seems that cannabis terpenoids and flavonoids, mainly myrcene, limonene, pinene, β-caryophyllene and cannflavin A, act in synergy with cannabinoids to induce pharmacological effects [35]. In fact, it was proven that these compounds, which are synthetized in the aerial parts of the plant, enhance CBD’s anti-inflammatory effects and antagonize THC dysphoric action [36,37].

Growing and storage may cause variations in the composition of herbal cannabis [38], making the interpretation of efficacy reported in clinical trials difficult. For these reasons, producers must carefully follow the WHO Good Agricultural and Collection Practices (GACP) for medical plants to obtain standardized products [39]. Furthermore, it is strictly recommended that flowering cannabis tops are stored in optimal, pre-established conditions. Indeed, when exposed to heat or light, carboxylated cannabinoids easily lose their carboxylic group, resulting in higher psychoactive cannabinoid rates [40]. It was demonstrated that one year of storage in darkness and at ambient temperature (approximately 25 °C), causes only slight changes in the content of THC and THCA-A, a significant decrease in CBD and a simultaneous increase in CBDA and CBC [38].

In Europe, the Office of Medical Cannabis Research (OMC), a Dutch government agency, became the first organization to obtain the exclusive right to supply medical cannabis to pharmacies and research institutes and, under the Single Convention on Narcotic Drugs of 1961, to import and export cannabis, cannabis extracts and cannabis resin for medical purposes [41]. OMC offers a variety of medical cannabis, all of which are dried female flowering tops, except for Bediol^®^, which is ground into small pieces to make its manipulation easier for patients with spasticity [42] (Table 1). These products can be also exported, with proper licenses, to other Member States of the European Union [41]. In Italy, to support cannabis cultivation for medical use and meet increasing public expectations [43], the Military Pharmaceutical Chemical Works of Florence (AID-SCFM) became the official national settlement for cultivating and manufacturing medical cannabis with a standardized cannabinoid content (decree of 9 November 2015) [44,45]. Two Italian products are currently available for consumers: FM1 and FM2, and their use was approved for the treatment of different forms of chronic pain and neurological disorders and other diseases resistant to standard therapies (Table 1). Outside Europe, the principal producers of medical cannabis are located in Canada. Specifically, Aurora Cannabis Inc. (ACB) [46] and Spectrum Therapeutics™ [47] offer a large variety of medical cannabis with different THC/CBD ratios (Table 1).

Cannabis female flowering tops can be simply administered through commercially available vaporizers (e.g., Micro Vape^®^, G Pen Herbal Vaporizer^®^ and Volcano^®^), buccal sprays (e.g., Sativex^®^) or oral capsules (e.g., Cannador^®^), decoctions or oils. To date, no specific guidelines for the preparation of medical cannabis decoctions or oils have been provided by any European or national pharmacopoeias [1].

### 3.1. Cannabis Flowering Tops Decoction Preparation

Following the indications given by Hazekamp et al. [48] and authorized by the Italian Ministry of Health [44], cannabis inflorescences should be immersed in a container containing cold water at a 0.1% concentration; it is recommended not to use less than 100 mL of water. The preparation should be heated to boiling point and simmered on a low heat for 15 min, while covered; it is recommended not to exceed 30 min in the heating of the decoction and to stir it at regular intervals. The decoction should be left to cool for approximately 15 min before filtering it. The filtrate should be stirred, then filtered on a strainer and the residue left on the filter should be pressed with a spoon to recover more liquid and enrich the final solution. 

It was demonstrated that the mean extraction recovery of cannabinoids in water is poor and variable. Recovery is approximately 18.5 + 8.6 and 13.8 + 4.7% for THC and THCA-A, respectively, and 28.1 + 10.3 and 58.2 + 21.1% for CBD and CBDA, respectively. These results highlight a significant decrease in the total THC/total CBD ratio from 0.71 in herbal matrices, to approximately 0.25 in the aqueous solution. Moreover, even when refrigerated at 4 °C, THC concentration in the decoction significantly decreased to less than 65% 3 days after preparation, while that of THCA-A and CBD decreased to less than 70% and 40%, respectively, after 7 days [34]. For this reason, extemporaneous preparation is recommended [48].

This preparation is the only one allowed in the above-mentioned Italian decree. On one hand, it is preferred by the patients because it is easy to prepare and presents a good safety profile, avoiding the psychotropic effect of accidental THC overdosing. On the other hand, since the extraction recovery is poor, a large volume of decoction is required to produce analgesic effects [48,49]. Therefore, a growing demand for oily preparations has risen, to make available a preparation that is easier to administer.

### 3.2. Cannabis Flowering Tops Oil Extract Preparation

Considering that the preparation of medical cannabis has never been described in any international pharmacopoeia, and that industrial provision is not yet available, pharmacies have been following Romano and Hazekamp’s indications from 2013 [34]. Although this study was the first to report this kind of preparation, a number of variants have been reported with no clear estimate of cannabinoid concentrations in the final product.

Therefore, a recent study proposed a standardized preparation method, clearly providing cannabinoid recovery and short- and long-term stability evaluations [34,38,40]. In this study, the extract is prepared with 500 mg of cannabis inflorescences in 5 mL of oil (olive oil, Ph.Eur). The product is heated in a bain-marie at approximately 98 °C for 120 min. The oil is cooled to room temperature and filtered. The extraction efficiency in oil is significantly higher and less variable than that in water, which is clearly explained by the lipophilic nature of cannabinoids [50]. THC and THCA-A recovery is approximately 62.4 + 0.1% and 61.8 + 9.2%, respectively, and CBD and CBDA recovery is 79.4 + 11.3% and 95.8 + 1.5%, respectively. Furthermore, even if the total THC/total CBD ratio (0.49) is lower than that of the flowering tops (0.71), it is much higher than that of the decoction (0.25). Interestingly, preheating flowering tops induces a total decarboxylation of acidic cannabinoids, resulting in an increased concentration of total cannabinoids in the final preparation [34]. Acidic precursors THCA-A and CBDA have been considered strategic for their pharmacological effects; thus, inflorescence preheating, although it increases THC and CBD concentrations, is questionable due to the scarce presence of acidic precursors. 

Regarding cannabinoid stability in oil preparations, when refrigerated at 4 °C in the dark, all substances lose their 25% initial concentration between 14 days and up one year after preparation. Evidently, this initial loss should be taken into consideration when prescribing the product [34,40].

### 3.3. Inhalation of Cannabis Flowering Tops with Vaporizing Systems

In the last ten years, the benefits of medical cannabis vaporization have become widely accepted. Commercial devices were developed to heat dried cannabis flowering tops to a temperature just below the combustion point (200 °C) in order to vaporize cannabinoids for inhalation with lower toxicity. A large variety of devices have flooded the market to meet patient needs. The most common commercially available vaporizers are Pen Vapes (e.g., Micro Vape from Vape), Portable Vaporizers (e.g., G Pen Herbal Vaporizer from Grenco Science) and Desktop Vaporizers (e.g., Volcano^®^ from Storz & Bickel) [51], which are easy to use and greatly improve patient compliance. However, there is currently no consensus on their safety profile. The preheating process converts acidic cannabinoids (THCA-A and CBDA) to their active decarboxylated forms (THC and CBD), but the conversion rate of each device is unknown. Moreover, the high quantity of THC inhaled could possibly lead to overdosing and psychotropic effects [52]. The long-term effects of cannabis vaping are currently unknown because vaporizers have not been commonly used for a long enough time to allow appropriate toxicological investigations [30]. However, it was reported that traces of additives in cannabis oil products can cause lung injuries when inhaled at a high temperatures, and several fatalities have been attributed to vaping devices [53,54]. Finally, there are not studies addressing the effects of vaporizing temperatures on the inhibition of fungal growth, and spores potentially present in cannabis flowering tops may produce infections in sick patients.

## 4. Vademecum for Prescribing Doctors

Although cannabis has been used for centuries for therapeutic purposes, there is currently no international law regulating cannabis use for specific treatments [49]. In US, cannabis is listed in schedule I of the Controlled Substances Act of 1970 [55,56]. However, many American states have legalized cannabis for medical and recreational purposes. Indeed, US physicians may release a “recommendation” [7], but cannot prescribe cannabis medicinal products, because they are not approved by the FDA and DEA and there is currently no medical use of cannabis medicinal products recognized by federal laws [57]. In Canada, the use of medical cannabis was approved in 1999 for the relief of oncologic pain. Canadians are authorized to possess herbal cannabis with a valid medical document containing information about the posology and validity of the treatment, under the Marihuana for Medical Purpose Regulations of 2014 [58]. Canada legalized cannabis recreational use in 2018, becoming one of the most important suppliers worldwide, especially in Europe. In the European Union, member states are free to establish their own national drug laws and several European countries such as the Netherlands, Germany, France, Romania and the Czech Republic have approved cannabis for medical use [59]. In Italy, medical cannabis use has been legislated since 2015, but only for the management of the chronic pain associated with multiple sclerosis and spinal medulla injury, for the control of nausea and vomiting due to chemotherapy, radiotherapy or HIV therapy, for the loss of appetite in oncologic and HIV-positive patients, for its stimulant appetite effect in cachexia and anorexia and its hypotensive effect in glaucoma and for the relieving of unintentional body and facial movements in Tourette syndrome when all conventional therapies are inefficient [44].

### 4.1. Medical Use and Physician’s Recommendations

This success of cannabis as medical product is principally due to the pharmacological properties of cannabinoids, mainly THC, CBD and their acid precursors, which involve a complex mechanism of signaling through the modulation of cannabinoid receptors CB_1_ and CB_2_ [60]. CB_1_s are predominantly expressed in the central and peripheral nervous system at the presynaptic terminals of neurons [24,61]. Their activation induces the presynaptic release of many neurotransmitters such as dopamine, *γ*-aminobutyric acid (GABA), glutamate, acetylcholine, serotonin and noradrenaline [18,26]. CB_2_s are principally expressed in peripheral tissues on the cells of the immune system [62,63]. CB_1_s activation in the cerebellum and the substantia nigra enhances the effect of GABA signaling, promoting muscle-relaxing in epileptic patients and reducing the spasticity and the unintentional movements of multiple sclerosis, Parkinson’s disease and Tourette syndrome [19,25,64]. Cannabinoids also act in the spinal cord, suppressing neuronal excitability through the hyperpolarization of neuronal membranes, and producing analgesia [65,66]. The latter effect is widely exploited for the management of chronic and neuropathic pain [67,68]. Moreover, the modulation of CB_1_s in the ciliary body dampens the presynaptic release of noradrenaline, reducing the intra-ocular pressure in glaucoma patients [69]. Apart from the effects involving CB_1_s and CB_2_s, cannabinoids interact with other cellular systems [36,70]. Indeed, through the modulation of 5-HT3 and 5-HT1A serotonin receptors, cannabis is used to treat nausea and vomiting in oncologic and HIV-patients when classical antiemetics are ineffective [71,72]. Moreover, oral, vaporized and smoked cannabis were shown to increase the release of ghrelin, a hormonal appetite stimulant, with positive effects in treating anorexia and cachexia [73,74]. In addition, CBD, terpenoids and flavonoids have neuroprotective and immunomodulatory effects, reducing chronic inflammation and decreasing oxidative damage [35,75]. For these reasons, cannabis has been proposed for the treatment of different forms of cancer, neurological and psychiatric disorders and inflammatory bowel diseases [75,76,77,78,79]. 

Despite cannabis possessing a tremendous potential in clinical practice, it is also a drug with a complex toxic profile and a high potential for abuse [56]. What makes cannabis attractive for recreational purposes is a series of positive mood effects due to the cannabinoid-mediated activation of dopaminergic neurons in the mesolimbic area [80]. This region is responsible for elaborating wellbeing and reward sensations, also representing a target for the development of the tolerance and dependence induced by other drugs that are also abused (e.g., alcohol, amphetamines) [81,82].

Cannabis pharmacological action is dose dependent and can induce many adverse effects (AEs), principally related to THC, due to unintentional overdosing [83]. The typical symptoms of cannabis acute intoxication are dizziness, confusion, tachycardia, postural hypotension, dysphoria, panic, depression and hallucinations [19,84,85,86]. Allergic reactions, vomiting and diarrhea are also reported [87]. While these AEs are transient and rapidly treated, a prescribing doctor must consider the risk/benefit ratio before administering medical cannabis to allergic individuals, patients with psychiatric disorders or patients treated with psychoactive drugs [88,89,90]. Chronic cannabis use is associated with an increased risk of developing substance use disorders (SUD), characterized by tolerance and dependence, which was demonstrated in animal models and has been reported in humans [24,91]. Furthermore, withdrawal symptoms like irritability, aggression, anxiety, insomnia, decreased appetite, tremors, sweating and headaches may appear after the abrupt cessation of the long-term administration of high doses of cannabis [92,93]. Therefore, it is not recommended to administer medical cannabis to patients with drug addictions or individuals with a history of drug addiction. Chronic cannabis use could also be a risk factor of steatosis in patients with severe hepatic impairment and chronic hepatitis [88,94,95].

Caution should also be observed with patients undergoing poly-drug therapy, especially the elderly and people with chronic diseases or kidney and liver impairments. Indeed, cannabinoids are extensively metabolized in the liver by different cytochrome (CYP) P450 isoforms such as CYP1A1/2, A4/5/7, 1B1, 2B6, 2C9/19, 2D6 and 2J2, and other enzymes involved in drug metabolism such as uridine diphosphate-glucuronyltransferase 1A9, 2B7 and carboxylesterase 1 [96,97,98]. Drug interactions with cannabinoids have mainly been reported for rifampicin, ketoconazole, warfarin, omeprazole, fentanyl, different antivirals, antiepileptics and chemotherapeutics [54,61,99]. In this regard, it is important for a physician to know the anamnestic history of the patient and his/her family in order to reduce the risk of treatment failure or drug toxicity.

Furthermore, despite the fact that cannabis vascular mechanisms are not well elucidated, cardiac AEs like bradycardia supraventricular tachycardia and palpitations have often been reported [83]. Therefore, in subjects with pre-existing cardiovascular illnesses, cannabis use could aggravate the symptoms of their diseases, leading to strokes and venous thromboembolic events [81].

Moreover, due to their high lipophilicity, cannabinoids can cross the blood/placenta and blood/breast milk barriers [100]. Stillbirth, growth retardation and impairment of fetal and postnatal cerebral development have been reported as a possible consequence of cannabis use during pregnancy or breastfeeding [89,90,95]. In addition, there is plenty of evidence showing that cannabis abuse during adolescence causes cognitive decline and poor attention and memory [59]. Therefore, a prescribing doctor should inform adults about these possible risks, also stressing the importance of keeping cannabis out of the reach of children, to avoid any unintentional ingestion [57,101]. 

Finally, a series of behavioral aspects should be considered for the health and the safety of the patient and the general population. First of all, only cannabis use through oral or inhalatory administration is allowed. Smoking reduces the bioavailability of cannabis ingredients by 40%, and complete combustion can cause serious lung diseases and airway obstruction [35,102,103]. Acute cannabis consumption is associated with a decrement in reaction time, motor coordination and attention [81]. Patients should be advised that their ability to drive or operate machinery could be impaired while they are under cannabis treatment [96].

### 4.2. *Dosages and Titration Strategies*

Even if cannabis is well tolerated and the majority of unwanted effects are mild and transient, there are some variables that do not fit well with the traditional medical model of drug prescription. First of all, there is no unique list of pathologies that can be treated with cannabis and it will be for the physicians to decide under which circumstances cannabis should be prescribed and which patients could benefit from the treatment [59]. Secondly, there are several strains of medical cannabis characterized by different percentages of THC and CBD (THC > CBD, THC ≅ CBD, THC < CBD), and every strain is associated to multiple pathological indications [5,60,78]:

- Anorexia: THC > CBD;
- Nausea and vomiting: THC > CBD;
- Insomnia: THC ≅ CBD;
- Depression: THC ≅ CBD;
- Pain: THC ≅ CBD;
- Multiple sclerosis: THC ≅ CBD;
- Tourette syndrome: THC ≅ CBD;
- Alzheimer’s disease: THC ≅ CBD;
- Anxiety: THC < CBD;
- Colitis: THC < CBD;
- Epilepsy: THC < CBD;
- Psychosis: THC < CBD;
- Parkinson’s disease: THC < CBD.

As a result, cannabis therapy is highly individualized. Cannabis dosages and titrations will be established by the prescribing doctor depending on the content of THC and CBD in herbal cannabis preparations, the route of administration and the risk/benefit ratio [50,102].

Oral administration has a lower bioavailability (5–20%) compared to inhalation, due to the gastric degradation of cannabinoids and first-pass hepatic metabolism [86]. Pharmacological effects range from 30 min to 3 h, lasting up to 12 h, while maximum cannabinoid blood concentration is reached within 2 h [89,104]. Despite this high time variability and low effect predictability, oral administration is recommended, since it is easy to prepare and self-administer. When oral administration does not produce any pharmacological effect, or when the physician considers it appropriate, it is possible to use vaporizers. Inhaled bioavailability varies from 10% to 35%, depending on the depth of inhalation, the puff frequency and the breath hold time. Cannabinoids’ pharmacological effects appear within a few minutes, and peak serum concentration is reached within 15 to 30 min with a maximal duration of 4 h [86,105]. In a similar manner to oral preparations, the prescribing doctor will indicate the quantity of flowering tops to use, the time interval and the number of daily inhalations.

For a long time, cannabis has not been treated as a pharmaceutical [58]. As a consequence, a standardized and universal regimen for medical cannabis titration has never been created. According to ICH efficacy and safety guidelines, it is recommended to start with low doses and increase quantities after a satisfactory period of clinic evaluation, depending on the pharmacological effects and possible AEs [106]. As medical cannabis toxicity occurs mainly due to THC, we will refer to THC equivalents for titration in this section.

The patient should start using a THC > CBD or a THC ≅ CBD preparation at bedtime to limit psychotropic AEs and development of tolerance, even though some subjects require THC for daytime use, depending on their symptoms [90,107,108]. It is recommended that between 1.25 and 2.5 mg THC equivalent is used once a day for the first two days and 2.5 mg THC equivalent is used twice a day during the third and fourth day. It is possible to increase the dose as needed and as tolerated to 15–20 mg THC equivalent two to three times during the therapy [108,109]. For cannabis inhalation, patients should start with one inhalation and wait 15 min; then, they may increase the dose by one inhalation every 15–30 min until the desired symptom control has been achieved [86,88]. Doses higher than 20–30 mg/day may increase AEs or induce tolerance without improving efficacy. In this concern, providing THC and CBD concentrations in oral cannabis preparations is essential for physicians to maximize efficacy and minimize safety risks in naive patients [107]. Therefore, using the data provided by Pacifici et al. [38,40], we calculated the total amount of THC and CBD in aqueous and oily preparations obtained from a variety of medical cannabis. The results are reported in Table 2. In any case, doses of THC-predominant cannabis above 5 g/day are probably unjustified, except in primary cancer treatments, and suggest possible tolerance or misuse [108].

## 5. Conclusions

Even though several cannabis-based pharmaceutical products are available, herbal preparations are still most widely used. However, the lack of standardized procedures for herbal preparations and the absence of posology protocols complicate the role of physicians in prescribing targeted therapies. In this review, the authors listed the most common procedures for the preparation of cannabis decoctions and oils and provided strategies to maximize the therapeutic potential and minimize the associated problems. The authors provided a practical and helpful guide for prescribing doctors with dosing and titration strategies and indicative THC and CBD amounts in aqueous and oily preparations, depending on the amount of product/extract and the variety of medical cannabis.

## Figures and Tables

**Table 1:** Herbal cannabis varieties for medical preparations.

Producer	Variety	%THC *	%CBD *	Origin	Indications
**THC > CBD**					Chronic pain; multiple sclerosis; nausea; vomiting; loss of weight; Tourette syndrome; glaucoma; cancer; epilepsy; inflammatory bowel diseases; Parkinson’s disease; psychiatric disorders.
Bedrocan International^®^	Bedrocan^®^	≅22	<1	Netherlands	
	Bedica^®^	≅14	<1		
	Bedrobinol^®^	≅13.5	<1		
Aurora Cannabis Inc. (ACB)	Pedanios 22/1	≅22	<1	Canada	
Spectrum Therapeutics™	Red n°1 Flower	20–23 (21.5 **)	<0.7		
	Red n°2 Flower	17–20 (18.5 **)	<0.7		
	Orange Flower	10–13 (11.5 **)	<0.7		
	Purple Flower	8–11 (9.5 **)	<0.7		
-	FM1	14.1	0.1	Italy	
**THC ≅ CBD**					
Bedrocan Inernational^®^	Bediol^®^	≅6.3	≅8	Netherlands	
Aurora Cannabis Inc. (ACB)	Pedanios 8/8	≅8	≅8	Canada	
Spectrum Therapeutics™	Blue Flower	6–9 (7.5 **)	8–11 (9.5 **)		
	Green Flower	4–7 (5.5 **)	7–10 (8.5 **)		
-	FM2	5.8	8.1	Italy	
**THC < CBD**					
Bedrocan International^®^	Bedrolite^®^	<1	≅9	Netherlands	
Aurora Cannabis Inc. (ACB)	Pedanios 1/9	<1	≅9	Canada	
Spectrum Therapeutics™	Yellow Flower	<1	11–14 (12.5 **)		

* delta-9-tetrahydrocannabinol (THC) and cannabidiol (CBD) percentages represent the sum of their decarboxylated (THC and CBD) and carboxylated forms (THCA-A and CBDA, respectively), which can be calculated using the following formulae [38,40]: %THC total = %THC + (0.877 X %THCA-A), %CBD total = %CBD + (0.877 X %CBDA), ** Mean percentages calculated from the range reported by the producer/seller.

**Table 2:** THC and CBD total amounts in aqueous and oily preparations *.

Varieties	Medical Cannabis Decoction						Medical Cannabis Oil					
	50 mL		100 mL		500 mL		10 Oil Drops		20 Oil Drops		50 Oil Drops	
	mg THC	mg CBD	mg THC	mg CBD	mg THC	mg CBD	mg THC	mg CBD	mg THC	mg CBD	mg THC	mg CBD
**THC > CBD**												
Bedrocan^®^	1.72	<0.30	3.43	<0.60	17.21	<2.30	4.37	<0.20	8.74	<0.40	21.84	<1
Pedanios 22/1	1.72	<0.30	3.43	<0.60	17.21	<2.30	4.37	<0.20	8.74	<0.40	21.84	<1
Red Flower n°1	1.68	<0.21	3.35	<0.42	16.81	<1.61	4.27	<0.14	8.54	<0.28	21.35	<0.70
Red Flower n°2	1.44	<0.21	2.87	<0.42	14.47	<1.61	3.67	<0.14	7.34	<0.28	18.36	<0.70
FM1	1.10	0.03	2.20	0.06	11.03	0.23	2.80	0.02	5.60	0.04	14.00	0.10
Bedica^®^	1.09	<0.30	2.19	<0.60	10.95	<2.30	2.78	<0.20	5.56	<0.40	13.90	<1
Bedrobinol^®^	1.05	<0.30	2.12	<0.60	10.55	<2.30	2.68	<0.20	5.36	<0.40	13.40	<1
Orange Flower	0.90	<0.21	1.79	<0.42	9.00	<1.61	2.28	<0.14	4.57	<0.28	11.42	<0.70
Purple Flower	0.74	<0.21	1.48	<0.42	7.43	<1.61	1.89	<0.14	3.77	<0.28	9.43	<0.70
**THC ≅ CBD**												
Pedanios 8/8	0.66	1.94	1.32	3.86	6.66	19.27	1.59	2.28	3.17	4.56	7.93	11.41
Blue Flower	0.62	2.30	1.24	4.59	6.25	22.88	1.49	2.71	2.97	5.42	7.44	13.55
Bediol^®^	0.52	1.94	1.04	3.86	5.25	19.27	1.25	2.28	2.50	4.56	6.25	11.41
FM2	0.48	1.96	0.96	3.91	4.83	19.51	1.15	2.31	2.30	4.62	5.75	11.55
Green Flower	0.46	2.06	0.91	4.10	4.58	20.47	1.10	2.42	2.18	4.85	5.45	12.12
**THC < CBD**												
Bedrolite^®^	<0.08	2.18	<0.17	4.34	<0.83	21.68	<0.20	2.57	<0.40	5.13	<1	12.83
Pedanios 1/9	<0.08	2.18	<0.17	4.34	<0.83	21.68	<0.20	2.57	<0.40	5.13	<1	12.83
Yellow Flower	<0.08	3.02	<0.17	6.03	<0.83	30.11	<0.20	3.56	<0.40	7.13	<1	17.82

* Reported amounts are indicative and depend on the above-reported variables: cannabinoids and precursors’ percentage in herbal products and extraction rates in aqueous and oily preparations.
